# Recent Advances in Detection, Isolation, and Imaging Techniques for Sulfane Sulfur-Containing Biomolecules

**DOI:** 10.3390/biom11111553

**Published:** 2021-10-20

**Authors:** Honami Echizen, Eita Sasaki, Kenjiro Hanaoka

**Affiliations:** 1 Graduate School of Pharmaceutical Sciences, The University of Tokyo, 7-3-1 Hongo, Bunkyo-ku, Tokyo 113-0033, Japan; echizen-honami761@g.ecc.u-tokyo.ac.jp; 2 Graduate School of Pharmaceutical Sciences, Keio University, 1-5-30 Shibakoen, Minato-ku, Tokyo 105-8512, Japan; e.sasaki@keio.jp

**Keywords:** sulfane sulfur, hydrogen sulfide, hydrogen persulfide, polysulfide, fluorescent probes

## Abstract

Hydrogen sulfide and its oxidation products are involved in many biological processes, and sulfane sulfur compounds, which contain sulfur atoms bonded to other sulfur atom(s), as found in hydropersulfides (R-S-SH), polysulfides (R-S-S_n_-S-R), hydrogen polysulfides (H_2_S_n_), etc., have attracted increasing interest. To characterize their physiological and pathophysiological roles, selective detection techniques are required. Classically, sulfane sulfur compounds can be detected by cyanolysis, involving nucleophilic attack by cyanide ion to cleave the sulfur–sulfur bonds. The generated thiocyanate reacts with ferric ion, and the resulting ferric thiocyanate complex can be easily detected by absorption spectroscopy. Recent exploration of the properties of sulfane sulfur compounds as both nucleophiles and electrophiles has led to the development of various chemical techniques for detection, isolation, and bioimaging of sulfane sulfur compounds in biological samples. These include tag-switch techniques, LC-MS/MS, Raman spectroscopy, and fluorescent probes. Herein, we present an overview of the techniques available for specific detection of sulfane sulfur species in biological contexts.

## 1. Introduction

Hydrogen sulfide (H_2_S) was shown to be a neuromodulator in the brain in 1996 [1], and since then, many biological studies have established that H_2_S is an endogenously produced gasotransmitter that is involved in multiple physiological processes, including cytoprotection against oxidative stress, mediation of neurotransmission, and regulation of inflammation [2]. In addition to H_2_S, reactive sulfur species produced by oxidation of H_2_S, including sulfane sulfur-containing molecules, play key roles in various biological processes [3,4,5]. Sulfane sulfur, often represented as S^0^, has an apparent oxidation state of zero, at least in one of its tautomeric forms, and is bonded to other sulfur atom(s) [6,7,8,9]. Sulfane sulfur-containing biomolecules include hydropersulfides (R-S-SH), hydropolysulfides (R-S-S_n_-SH), polysulfides (R-S-S_n_-S-R), etc. Cysteine/glutathione persulfides or polysulfides have physiological roles in antioxidation and cytoprotection [10]. Hydrogen polysulfides (H_2_S_n_) are involved in the activation of the channels for Ca^2+^ influx in astrocytes and dorsal root ganglion neurons [11]. Additionally, excess production of H_2_S and polysulfides underlies the pathophysiology of schizophrenia [12]. To understand the biological roles of sulfane sulfur-containing biomolecules, chemical techniques that can specifically detect sulfane sulfur compounds in biological samples are essential. Sulfane sulfur reacts with cyanide ion (CN^−^) to form thiocyanate (SCN^−^), which is easily detected by absorption spectroscopy following a reaction with ferric ion [6]. However, this classical method cannot be applied for isolation and identification of sulfurized proteins in biological samples or for bioimaging of sulfane sulfur compounds in cells and tissues. Recently, the unique chemical properties of sulfane sulfur compounds as nucleophiles and electrophiles have been explored [8,13], leading to the development of new methods of specific detection. Here, we review current techniques available for detection, isolation, and imaging of these compounds in biological samples.

### Tag-Switch Techniques

**Figure 1:**
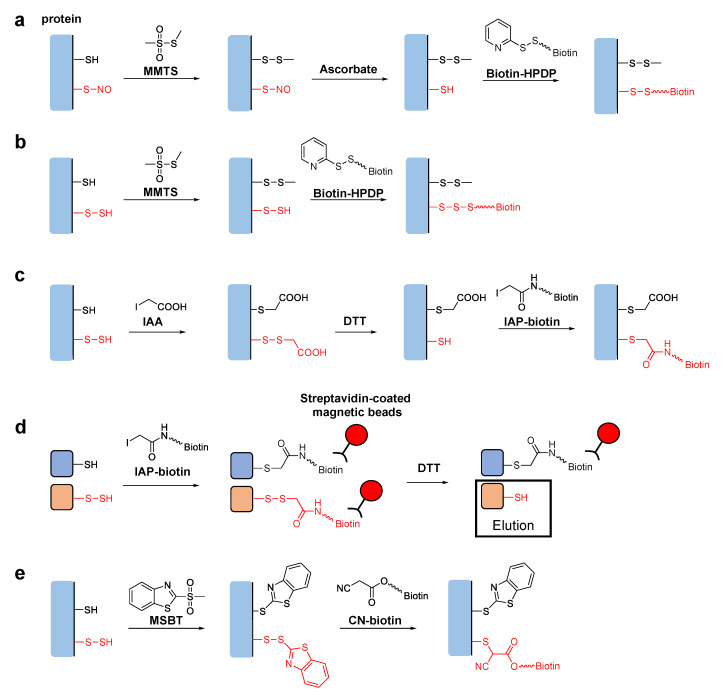
(**a**) Proposed mechanism of the tag-switch technique for the detection of nitrosothiol modification in proteins. (**b**–**d**) Proposed mechanisms of tag-switch techniques for the detection of cysteine per/polysulfides in proteins. The target persulfide groups are switched to biotin-linked modifications derived from (**b**) biotin-HPDP and (**c**) IAP-biotin for further analyses. (**d**) To isolate persulfurized proteins, the biotin-linked disulfides derived from IAP-biotin can be selectively reduced by DTT and eluted from the streptavidin-coated magnetic beads. (**e**) CN-biotin can also be used to switch the target persulfide groups to biotin-linked modifications for further analyses.

Tag-switch techniques are used to identify specific post-translational modifications of proteins. The original tag-switch technique, also known as the biotin switch assay, was developed for detection of *S*-nitrosylation, i.e., the post-translational modification of cysteine residues with nitric oxide (NO) [14,15]. To detect *S*-nitrosylated proteins, free thiol groups are first blocked with methyl methane thiosulfonate (MMTS). The target *S*-nitrosothiol (-SNO) groups are then reduced with ascorbate to generate new thiol groups, which are specifically biotinylated with a pyridyldisulfide-containing biotinylation reagent, *N*-[6-(biotinamido)hexyl]-3′-(2′-pyridyldithio)propionamide (biotin-HPDP) (Figure 1a). The biotinylated proteins can be isolated on a streptavidin column for further analyses, such as sodium lauryl sulfate polyacrylamide gel electrophoresis (SDS-PAGE), immunoblotting, etc.

Based on the analogy of the biotin switch assay for *S*-nitrosylation, several new techniques have been developed to detect persulfurized cysteine residues in proteins. Mustafa et al. reported that the sequential addition of MMTS and biotin-HPDP without going through the reduction step with ascorbate could specifically biotinylate persulfurized proteins in mouse liver (Figure 1b) [16]. They claimed that MMTS reacted with thiols but not with persulfides in the first step, although the reason for this selectivity was unclear. Later studies showed that persulfide groups could also react with MMTS, raising an issue concerning the underlying mechanism proposed in the original report [17]. Krishnan et al. reported another biotin switch technique using iodoacetic acid (IAA) [18]. IAA reacts with both thiol and persulfide groups, but subsequent dithiothreitol (DTT) treatment reduces only disulfide bonds of the persulfide-derived products. This generates new thiol groups, which are specifically labeled with iodoacetyl-PEG_2_-biotin (IAP-biotin) (Figure 1c). Although this method can distinguish persulfide groups from thiol groups in theory, a potential limitation is that other cysteine modifications, including disulfide bonds and *S*-nitrosylation, could also be detected because they are reduced by DTT and react with IAP-biotin as well [17]. More recently, Dóka et al. reported a method using IAP-biotin in the first step to react with both thiol and persulfide groups [19]. In their protocol, biotinylated proteins were trapped on streptavidin-coated magnetic beads. Thus, proteins originally possessing persulfide groups could be selectively eluted after reducing the disulfide bonds to liberate the biotin moiety (Figure 1d). Using this technique, they quantified protein persulfide concentrations in cultured cells and mouse liver. Notably, this method is not affected by other oxidative modifications of cysteine residues, such as disulfide bonds and *S*-nitrosylation, because they either do not react with IAP-biotin or may react but do not generate biotinylated cysteine products having DTT-sensitive disulfide linkages. Zhang et al. also reported a selective tag-switch method for persulfide groups [20]. They first developed a new thiol blocking reagent, methylsulfonyl benzothiazole (MSBT) [21]. MSBT reacts with both thiols and persulfides to generate products with thioether and disulfide linkages, respectively. They found that the enhanced reactivity of the disulfide bonds in the MSBT-labeled persulfides enabled efficient attack by biotin-linked cyanoacetate (CN-biotin), leading to specific biotinylation (Figure 1d) [20].

In the tag-switch assays for persulfurized proteins mentioned above, per/polysulfides can be selectively labeled with biotin for subsequent detection and/or isolation of the proteins by using streptavidin-linked materials, such as a streptavidin column, streptavidin-coated magnetic beads, streptavidin-horseradish peroxidase conjugate, etc. It is also possible to perform in situ fluorescence imaging of intracellular *S*-sulfhydration with fluorophore-labeled streptavidin. The same tag-switch chemistry is also readily applicable to covalently link fluorophores instead of biotin with the target cysteine residues of the persulfurized proteins [22,23].

## 2. LC-MS/MS

**Figure 2:**
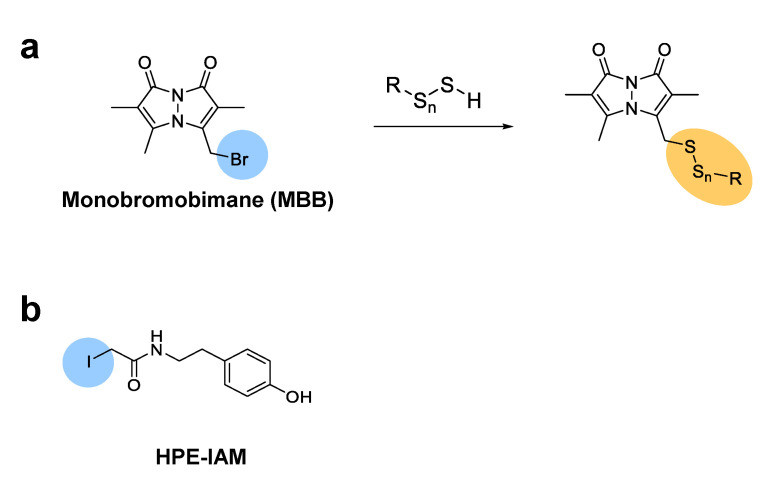
Chemical structures of MBB (**a**) and HPE-IAM (**b**), alkylating agents for LC-MS/MS analysis of hydropersulfides and hydropolysulfides.

For the detection of persulfides and polysulfides in biosamples by liquid chromatography-tandem mass spectrometry (LC-MS/MS), samples are first alkylated with alkylating reagents such as monobromobimane (MBB). As shown in Figure 2a, hydropersulfides and hydropolysulfides react with MBB, and the adducts can be analyzed by HPLC with a scanning fluorescence detector and tandem MS [24]. For example, Ida et al. analyzed the levels of various polysulfides in mouse heart by LC-MS/MS [10]. They quantified the MBB-modified persulfides and polysulfides, such as cysteine persulfide (CysSSH), glutathionine persulfide (GSSH), glutathionine polysulfide (GSSSG), etc., and found that these per/polysulfide molecules were endogenously produced and maintained in the plasma and cells. However, the strong electrophilicity of MBB may be problematic because it may lead to decomposition of the polysulfide adducts during the alkylation process [25]. Related to this issue, sulfide detection with MBB in biological samples such as human blood serum and plasma requires special caution because MBB irreversibly consumes free sulfide and continues to extract sulfide from other reversibly bound sulfide complexes [26]. Thus, another alkylating reagent, β-(4-hydroxyphenyl)ethyl iodoacetamide (HPE-IAM), with milder electrophilicity was used to form stable adducts without causing decomposition of hydropolysulfides (Figure 2b) [27]. Different reactivity of MBB and HPE-IAM toward per/polysulfide species was carefully examined, and it was confirmed that HPE-IAM does not efficiently cleave dialkyl polysulfide chains [25]. Recently, Akaike et al. successfully used HPE-IAM to precisely quantify various hydroper/polysulfides in mammalian cells. They established that cysteinyl-tRNA synthetase biosynthesizes cysteine hydropersulfide and is involved in the electron transport chain in mitochondria [28]. It is now generally believed that cysteinyl-tRNA synthetase has a role in translation-coupled protein *S*-sulfhydration [29].

Among many sulfane sulfur species, cyclic oligosulfurs such as S_8_ are difficult to analyze with MS because uncontrollable fragmentation usually occurs. To address this issue, Matsuno et al. recently reported a synthetic polyaromatic capsule, which encapsulates cyclic oligosulfur species based on host–guest chemistry [30]. They demonstrated that spontaneous encapsulation of S_8_ in the capsule occurred in water at room temperature. The host–guest structure was verified by NMR and X-ray crystallographic analysis. Notably, this complex can be detected by common electrospray ionization time-of-flight MS as the intact form. So far, the existence of cyclic oligosulfur clusters in biological samples such as cells and tissues has been largely unknown because of a lack of an appropriate analytical method. Thus, this method may become a useful tool to investigate the importance of cyclic oligosulfurs in biological contexts in the future.

## 3. Raman Imaging

Surface-enhanced Raman spectroscopy (SERS) is a non-invasive analytical technique with high sensitivity in the presence of reporter metals such as gold and silver. Raman signals of analytes are enhanced because the excitation of surface-plasmon resonance amplifies the local electromagnetic field. SERS has been used for many applications, including trace-molecule detection and biosensing [31]. It has also been used for biological imaging at the single cell level or in ex vivo samples [32,33]. Recently, Shiota et al. detected Raman signals of endogenous polysulfides in brain tissues by using random arrays of horse-bean-shaped gold nanostructures, called gold nanofève (GNF) [34]. GNF generates many electromagnetic hotspots and enables large-area visualization of analytes. They applied this technique to bioimaging of metabolites in a mouse syngeneic model of glioblastoma and found that endogenous polysulfide compounds could be visualized as a peak at 480 cm^−1^. More recently, Honda et al. used the same SERS technique to identify sulfur-containing metabolites associated with chemoresistance mechanisms of certain ovarian cancers [35]. They observed the SERS signal at 480 cm^−1^ in clear cell carcinoma, which suggests that polysulfides are present in the cancer tissues. Further development of Raman imaging techniques for sulfane sulfur compounds may lead to a deeper understanding of malignancies, as well as providing tools for predicting the efficacy of chemotherapy.

## 4. Fluorescent Probes

Fluorescent probes are synthetic small molecules or proteins that emit fluorescence upon reaction with the target analytes. They are easy to use and potentially useful for real-time imaging in living cells and tissues. The fluorescence switching mechanisms of these probes rely on the high nucleophilicity of per/polysulfides and the ability of sulfane sulfur to reversibly form a bond with another sulfur atom.

A few protein-based fluorescent probes for sulfane sulfur-containing biomolecules have been developed. A recently reported polysulfide-sensitive green fluorescent protein (psGFP) is a prominent example [36]. In this probe, a pair of cysteine residues was introduced to form internal polysulfide bonds in the presence of sulfane sulfur sources. Since the internal polysulfide bond alters the equilibrium of the GFP chromophore between the neutral and anionic forms, which have characteristic excitation properties, it is possible to estimate the proportion of oxidized (polysulfurized) and reduced psGFP by analyzing the excitation spectra. Protein-based fluorescent probes may have advantages over small molecule-based probes in at least one respect, such as ease of localization in subcellular organelles by simply fusing targeting signal peptides, however, only a few biological applications using the protein-based polysulfide probes with model microorganisms have been demonstrated so far. Thus, we focus on reviewing the molecular design and fluorescence switching mechanisms of small molecule-based sulfane sulfur probes and their biological applications in the following part of this section.

**Figure 3:**
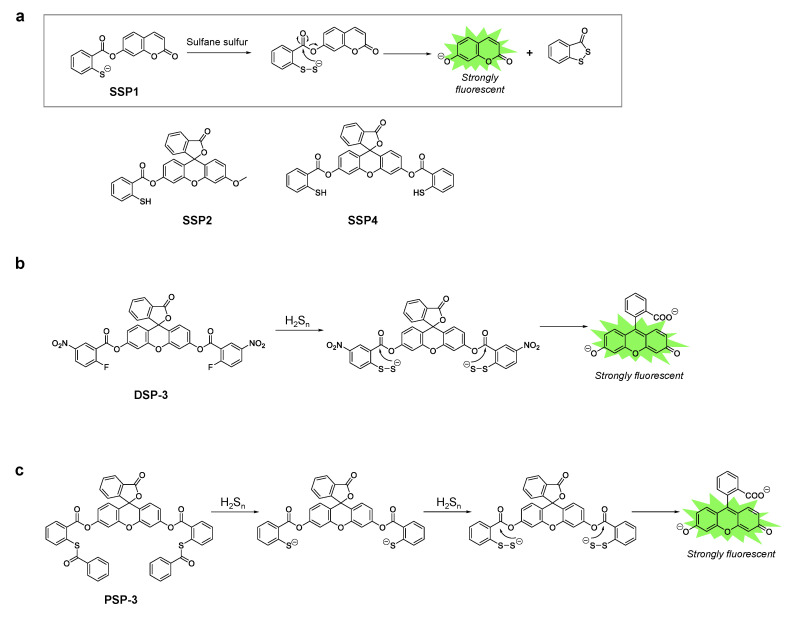
(**a**) Chemical structures of the SSP series and the proposed reaction mechanism with sulfane sulfur. (**b**) Chemical structure of DSP-3 and its reaction mechanism with hydrogen polysulfides. (**c**) Chemical structure of PSP-3 and its reaction mechanism with hydrogen polysulfides.

A series of fluorescent probes for sulfane sulfur, called SSP, was first developed by Xian’s group [37]. SSP1 and SSP2 have a coumarin scaffold and a fluorescein scaffold respectively, and initially lack fluorescence. Both probes contain a thiol group at which sulfane sulfur reacts to form benzodithiolone. This reaction intermediate undergoes spontaneous intramolecular cyclization to release the fluorophore, which generates a strong fluorescence signal (Figure 3a). Upon reaction with a persulfide donor (Na_2_S_2_), SSP1 and SSP2 showed 25- and 50-fold fluorescence enhancements, respectively. In addition, both probes have high selectivity for sulfane sulfur, however they did not show significant fluorescence enhancement in the presence of other sulfur species such as cysteine, glutathione (GSH), H_2_S, etc. The probes could also be applied to detect sulfane sulfur in cells. For instance, SSP4, which has a fluorescein scaffold with two reaction sites for sulfane sulfur (Figure 3a), has been widely used for intracellular imaging of sulfane sulfur species. To load the probe, the cells were only incubated with the probe in DMEM containing a cationic surfactant, CTAB, or a non-ionic surfactant such as cremophor EL or Pluronic F127 [38,39,40].

Xian’s group also reported fluorescent probes for hydrogen polysulfides called DSP and PSP [41,42]. The DSP series contains a 2-fluoro-5-nitrobenzoic ester moiety at which nucleophilic aromatic substitution occurs with highly nucleophilic hydrogen polysulfides (Figure 3b) [41]. The reaction intermediate containing the persulfide group spontaneously cyclizes with concomitant release of the fluorophore, and this results in a strong fluorescence enhancement. In the case of PSP, thioesters react with hydrogen polysulfides [42]. Cleavage of two thioesters from PSP-3 leads to the same structure as SSP4, which further reacts with hydrogen per/polysulfide and becomes fluorescent, and the mechanism is the same as that described above for SSP (Figure 3c).

**Figure 4:**
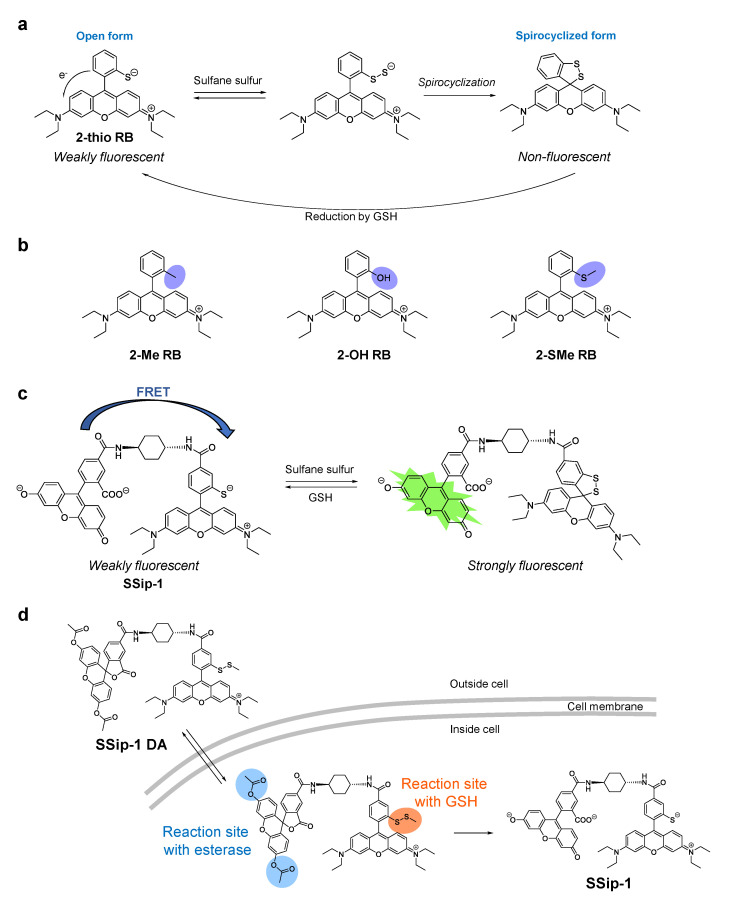
(**a**) Chemical structure of 2-thio RB and proposed mechanism of its reaction with sulfane sulfur and reduction by GSH. (**b**) Chemical structures of 2-Me RB, 2-OH RB, and 2-SMe RB. (**c**) Chemical structure of SSip-1 and the proposed reaction mechanism with sulfane sulfur. (**d**) Chemical structure of SSip-1 DA designed for live-cell imaging of sulfane sulfur. SSip-1 DA is expected to have high membrane permeability and to react with intracellular esterase and GSH to release SSip-1.

While the SSP, PSP, and DSP series of sulfane sulfur fluorescence probes show an irreversible fluorescence enhancement with sulfane sulfur compounds, we have recently developed a reversible fluorescent probe called SSip-1 to monitor the intracellular dynamics of sulfane sulfur [40]. To achieve the reversible fluorescence response, SSip-1 was designed to utilize the formation of sulfane sulfur adducts with thiols and the intramolecular spirocyclization reaction of xanthene dyes. Specifically, we selected 2-thiorhodamine B (2-thio RB) to react with sulfane sulfur (Figure 4a), and used this moiety as a fluorescence off/on modulator, as described below.

When sulfane sulfur forms an adduct at the thiol group of 2-thio RB, spontaneous intramolecular spirocyclization occurs. This reaction results in a loss of absorption in the visible region due to disruption of the π conjugation between two aromatic rings of the fluorophore. Indeed, we found that the absorbance of 2-thio RB decreased upon the addition of a sulfane sulfur donor, Na_2_S_4_. In addition, MS analysis of the reaction mixture revealed the existence of the spirocyclized form of 2-thio RB with a sulfane sulfur. To confirm that the 2-thio group is essential for the reaction, 2-Me, 2-OH, and 2-SMe derivatives of rhodamine B were reacted with Na_2_S_4_ in sodium phosphate buffer (Figure 4b). As expected, none of these compounds showed any absorption spectral change. We also examined the reactivity of 2-thio RB with sulfane sulfurs in the presence of a biologically relevant reductant, GSH, at the concentration of 5 mM, since GSH is reported to be present at the mM level in living cells [43]. The absorbance of the reaction mixture initially decreased even in the presence of GSH, but gradually recovered. This suggests that GSH reduced the disulfide bond of the spirocyclized form of the 2-thio RB-sulfane sulfur adduct to generate the open form of 2-thio RB (Figure 4a). Thus, a reversible absorbance response of 2-thio RB upon addition and removal of sulfane sulfur was confirmed. However, the dynamic range of fluorescence intensity during the process is rather small, because 2-thio RB is only weakly fluorescent, likely due to fluorescence quenching via intramolecular photoinduced electron transfer (PeT) (Figure 4a) [44]. Therefore, we considered using 2-thio RB as a quencher of another fluorescent dye so that it would modulate the fluorescence signal in proportion to the progress of the reaction with sulfane sulfur. Specifically, 2-thio RB and fluorescein were connected via a cyclohexyl linker (Figure 4c). We expected that Förster resonance energy transfer (FRET) from fluorescein to 2-thio RB would occur because of the large overlap of the fluorescence spectrum of fluorescein with the absorption spectrum of 2-thio RB. Indeed, SSip-1 was weakly fluorescent before the reaction with sulfane sulfur, but the fluorescence at 525 nm increased within 1 min upon addition of Na_2_S_4_. Furthermore, repeated cycles of Na_2_S_4_ and GSH addition demonstrated that the response of SSip-1 to sulfane sulfur is reversible. SSip-1 showed fluorescence enhancement in the presence of hydrogen per/polysulfide donors, Na_2_S_2_, Na_2_S_3_, and Na_2_S_4_, but the addition of other sulfur species such as cysteine, GSH, and Na_2_S resulted in no fluorescence increase. Thus, the reaction was confirmed to be specific for sulfane sulfur species. 

We also developed a cell-membrane-permeable sulfane sulfur probe called SSip-1 DA, which is a diacetylated and thiol-protected derivative of SSip-1 (Figure 4d). We expected that the acetyl groups would be hydrolyzed, and the disulfide bond would be reduced by intracellular esterase and GSH respectively, when the probe is located in cytosol. Indeed, SSip-1-loaded A549 cells showed an intracellular fluorescence increase upon addition of Na_2_S_4_. Further incubation resulted in a decrease of the fluorescence intensity over 30 min, reflecting the reducing environment in the cytosol, but rapid fluorescence enhancement was again observed upon the second addition of Na_2_S_4_. This detection cycle could be repeated at least three times, indicating that SSip-1 can reversibly detect hydrogen polysulfide in living cells.

SSip-1 DA also worked in primary-cultured hippocampal astrocytes. Miyamoto et al. used this probe to investigate the effect of polysulfides on the transient receptor potential ankyrin 1 (TRPA1) channel, and found that hydrogen per/polysulfides were generated by the interaction of H_2_S and NO [11]. They examined the levels of hydrogen per/polysulfide production using Na_2_S (H_2_S donor) and DEA/NO (NO donor) in the presence or absence of reducing substances in dorsal root ganglion neurons. The results suggested that hydrogen polysulfides may be synergistically generated by the interaction of H_2_S and NO to activate TRPA1 channels in cells.

Finally, it should be noted that fluorescent probes are useful not only for bioimaging, but also for high-throughput screening of selective inhibitors of enzymes, if the enzymatic reaction products can be sensed by the probes. Cystathionine *γ*-lyase, cystathionine *β*-synthase, 3-mercaptopyruvate sulfurtransferase (3MST), and cysteinyl-tRNA synthetase are H_2_S- and/or sulfane sulfur-containing biomolecule-producing enzymes in humans [8,10,39,45], so fluorescent probes that detect H_2_S or its oxidized products are potentially useful for finding effective inhibitors of these enzymes. We recently discovered several potent inhibitors of 3MST by means of high-throughput screening of a large chemical library (over 170,000 compounds) using our H_2_S-selective fluorescent probe HSip-1 [46,47]. Interestingly, one of the inhibitors was confirmed to be highly specific to 3MST, and the crystal structure of the complex revealed that it binds near a persulfurated cysteine residue at the active site of 3MST.

## 5. Conclusions

Here, we have briefly reviewed detection techniques for sulfane sulfur-containing biomolecules such as persulfides and polysulfides. Further development of these methods is needed to visualize the dynamics of reactive sulfur species in living cells or tissues. In addition, combinations of different detection techniques would be useful to uncover the physiological and pathophysiological roles of reactive sulfur species in redox biology. Consequently, research to improve detection technologies for these sulfur compounds is currently a hot topic.
